# The gas production, ruminal fermentation parameters, and microbiota in response to *Clostridium butyricum* supplementation on *in vitro* varying with media pH levels

**DOI:** 10.3389/fmicb.2022.960623

**Published:** 2022-09-21

**Authors:** Meimei Zhang, Gege Liang, Xinlong Zhang, Xiaotan Lu, Siyao Li, Xu Wang, Wenzhu Yang, Yuan Yuan, Peixin Jiao

**Affiliations:** ^1^College of Animal Science and Technology, Northeast Agricultural University, Harbin, China; ^2^College of Food Science, Northeast Agricultural University, Harbin, China; ^3^Lethbridge Research and Development Centre, Lethbridge, AB, Canada; ^4^School of Nursing and School of Public Health, Yangzhou University, Yangzhou, China

**Keywords:** *Clostridium butyricum*, batch culture, media pH level, microbiota, rumen fermentation

## Abstract

The aim of this study was to investigate the gas production (GP), dry matter disappearance (DMD), fermentation parameters, and rumen microbiota in response to *Clostridium butyricum* (CB) supplementation in batch culture using a high forage substrate. The doses of CB were supplemented at 0 (Control), 0.5 × 10^6^, 1 × 10^6^, and 2 × 10^6^  CFU/bottle, respectively, at either media pH 6.0 or pH 6.6. The 16S rRNA gene sequencing was used to detect the microbiota of fermentation culture in control and 1 × 10^6^  CFU/bottle after 24 h of incubation. The results showed that the GP (*p* < 0.001), DMD (*p* = 0.008), total volatile fatty acid (VFA) concentration (*p* < 0.001), acetate to propionate ratio (*p* < 0.001), and NH_3_-N concentration (*p* < 0.001) were greater at media pH 6.6 than pH 6.0. Furthermore, the linearly increased DMD (pH 6.0, *p* = 0.002; pH 6.6, *p* < 0.001) and quadratically increased butyrate proportion (pH 6.0, *p* = 0.076; pH 6.6, *p* < 0.053) and NH_3_-N concentration (pH 6.0, *p* = 0.003; pH 6.6, *p* = 0.014) were observed with increasing doses of CB. The Alpha diversity indexes of OTU number and Chao1 were higher (*p* = 0.045) at media pH 6.6 than pH 6.0, but they were not affected by CB supplementation. The PCoA analysis (unweighted uniFrac) demonstrated that the clustering of the bacterial microbiota of control and CB were distinctly separated from each other at media pH 6.0. At the phylum level, the abundance of *Bacteroidota* (*p* < 0.001) decreased, whereas that of *Firmicutes* (*p* = 0.026) increased when the media pH was elevated from 6.0 to 6.6. Supplementation of CB increased relative abundances of *Rikenellaceae_RC9_gut_group* (*p* = 0.002), *Christensenellaceae_R-7_group* (*p* < 0.001), and *NK4A214_group* (*p* = 0.002) at genus level. Interactions between media pH and CB addition were observed for bacteria at both phylum and genus levels. These results indicated that increasing the media pH level and CB supplementation increased *in vitro* rumen digestibility, and altered the ruminal fermentation pattern (by media pH) and microbiota.

## Introduction

The use of antibiotics has contributed to the improvement of animal production and animal health for several decades. Effective antibiotic growth promoters have been commonly fed to livestock to improve feed efficiency and animal health (Lana et al., 1997; Ítavo et al., 2011). However, there are various concerns involving the use of antibiotics including environmental pollution, food safety issues, and risks of developing antibiotic resistance in microbes associated with animal or human diseases. Consequently, discussion around the potential ban of antibiotics has become a trend, but this would impose a considerable challenge for livestock production. Hence, it is crucial to develop economically competitive alternatives (e.g., probiotics, prebiotics, or plant extracts) to antibiotics to meet the requirements of improving animal health and products (Jiao et al., 2017, 2021).

Probiotics, defined as “live microorganisms,” have been considered as potential alternatives to antibiotics to improve growth performance and immunity of pigs (Roselli et al., 2017), sheep (Mani et al., 2021), beef cattle (Ran et al., 2018), or dairy cows (Chiquett et al., 2012). In ruminants, probiotics are considered oxygen consumers or nutrient providers to microorganisms in the rumen for the modulation of ruminal fermentation, and inhabitation of detrimental microorganisms by producing some antimicrobial substances like bacteriocins (Chaucheyras-Durand et al., 2008; McAllister et al., 2011), thereby improving growth performance. Our previous studies confirmed the beneficial effect of probiotics with yeast or lactic acid bacteria on stimulating ruminal fermentation and increasing the copy numbers of cellulolytic bacteria. However, the effects of probiotics varied considerably among different experimental studies, and these differences are likely due to a number of factors that can impact the final outcomes, such as type of probiotics, supplementation dose, media pH level, and type of substrate used (Jiao et al., 2017, 2018, 2019).

As a gram-positive anaerobe, *Clostridium butyricum* (CB) is known as a producer of butyric acid that forms spores, and is widely used in monogastric feeding to improve growth performance, feed efficiency, and animal health (Wang et al., 2020; Zhang et al., 2020). Because CB can survive in low pH and high bile concentrations, previous studies mainly focused on its role in the modulation of gut microbial composition and improvement of intestinal health (Cao et al., 2019; Li et al., 2019). In ruminants, a recent study conducted by Li et al. (2021) showed that dietary supplementation of CB improved growth performance and enhanced ruminal fermentation by increasing the abundance of rumen microbiota and molar proportion of propionate and butyrate in Holstein heifers. The common diet of lactating dairy cows usually involves highly fermatable concentrate and finely chopped silage (i.e., low physical effectiveness) to meet the energy requirement for high milk production. Therefore, low rumen pH (<5.8) could be observed, particularly following feed ingestion. Similarly, Cai et al. (2021a) reported that the CB supplementation increased total VFA concentration and improved growth performance of heat-stressed goats. Although those studies (Cai et al., 2021a; Li et al., 2021) have investigated the effects of CB supplementation on rumen fermentation characteristics, ruminal pH was at a high level (≥6.5), and limited information on rumen microbiota was obtained. We have identified a research gap when it comes to the impact of CB supplementation on gas production (GP) and rumen microbiota, particularly with varying supplementation doses and ruminal pH level. We hypothesized that supplementing *C. butyricum* would increase dry matter disappearance (DMD) and rumen fermentation characteristics, and improve rumen microbiota at both phylum and genus levels, depending on media pH level and supplemental dose. Hence, the objective of this study was to evaluate the effects of CB supplementation varying with media pH levels (6.0 or 6.6) and supplemental doses on gas production (GP), fermentation parameters, and microbiota in batch culture.

## Materials and methods

### *Clostridium butyricum* resource

The CB strain evaluated in this study was obtained from Greensnow Biological Biotechnology Co., Ltd. (Wuhan, China). The concentration of the CB sample was 2.0 × 10^8^ CFU/g.

### Experimental design, substrate, and inoculum

The experiment was conducted using a completely randomized design with two media pH levels (6.0 and 6.6) × 4 supplemental dosages of CB at control (0 CFU/bottle), low (0.5 × 10^6^ CFU/bottle), medium (1 × 10^6^ CFU/bottle), and high (2 × 10^6^ CFU/bottle). The media pH of 6.0 and 6.6 were chosen based on the averaged rumen pH of dairy cow diet (Palmonari et al., 2010). The supplemental doses of CB were outlined according to the manufacturer’s recommendations. A typical dairy cow diet was used as substrate (Table 1). Rumen inoculum was provided by two ruminally fistulated nonlactating dairy cows fed a diet based on 55% forage and 45% concentrate (DM basis). A total mixed ration was prepared daily and offered twice at 06:00 h in the morning and 18:00 h in the afternoon. All experimental procedures involving animals were performed according to the experimental license (protocol number: NEAU-[2011]-9) of Northeast Agricultural University (Harbin, China).

**Table 1:** Ingredients and chemical composition of experimental diets.^a^

Item	Content
**Ingredients, %**	
Alfalfa hay	28.38
Corn Silage	26.86
Soybean meal	15.6
Expanded soybean	2.69
Ground Corn	19.48
Double low rapeseed meal	3.76
Premix^b^	3.23
**Chemical composition, % of DM**	
DM, %	75.8
Organic matter	92.8
Crude protein	16.4
Ether extract	3.9
Neutral detergent fibre	35.2
Acid detergent fibre	18.9
Calcium	0.86
Phosphorus	0.43
NE_L_ (MJ/kg)^c^	7.02

a The diet was also used to feed rumen inoculum donor.

b Premix contained (per kilogram): 1,500 mg of Fe, 1,500 mg of Mn, 800 mg of Cu, 30 mg of I, 2,000 mg of Zn, 60 mg of Co, 80 mg of Se, 300,000 IU of VA, 100,000 IU of VD_3_, and 2,000 IU of VE.

c The NE_L_ was estimated using National Research Council (NRC) (2001).

### Batch culture procedures

The batch culture was conducted using 125 ml glass bottles, and rubber stoppers and aluminum caps were used to seal the bottles. Approximately 0.5 g (DM basis) of ground substrate was passed through a 1-mm screen and weighed into the acetone-washed and preweighed filter bags (F57; Ankom Technology, Macedon, NY, United States). The CB samples were added into the bottles in three replicates at desired dosages. Rumen contents were collected 2 h before the morning feeding from four different locations within the rumen, composited and squeezed through a nylon mesh. The strained rumen fluid was kept in an insulated air tight container. The rumen fluid was re-strained through four layers of cheesecloth to remove particles, and warmed at 39°C in a water bath in the laboratory. The pH of rumen fluid was 6.74, 6.81, and 6.69 for the first, second, and third run, respectively. Forty-five milliliters of freshly prepared warm buffer and 15 ml of strained rumen fluid were added into each bottle with continuously flushing of carbon dioxide into the bottle to remove the air from the headspace. The bottles were sealed with rubber stoppers and aluminum caps, and then transferred to a shaking incubator (SPH-2102C, Shanghai Huyueming Scientific Instrument Co., Ltd., Shanghai, China) at 39°C for 24 h. The two media pH levels (6.0 and 6.6) were achieved according to the method described by Jiao et al. (2019) that was adjusted to the volume of sodium bicarbonate in the buffer solution. At each media pH level, three additional bottles containing the same volume of buffer and rumen fluid without substrate or CB additive were used as blanks for correcting GP due to fermentation media. The same batch culture was repeated weekly for another two runs and a run was used as an experimental replicate.

Gas pressure was recorded at 3, 6, 9, 12, and 24 h of incubation using a pressure transducer (model HT-935, Hongcheng Technology, Shenzhen, China) with a 23-gauge needle (0.6 mm) passing through rubber stoppers. Pressure values, corrected for the amount of substrate DM incubated and the gas released from the blanks, were used to generate gas volume (GV) using equation of Romero-Perez and Beauchemin (2018): GV = 4.7047 × (gas pressure) + 0.0512 × (gas pressure^2^). After 24 h of incubation, the bottles were placed in cold water to stop fermentation. The pH of the fermentation fluid was measured using a portable pH meter (PHS-3C; Nanjing Nanda Analytical Instrument Application Research Institute, Nanjing, China) after removing the aluminum caps and rubber stoppers. Samples of 5 ml of fermentation fluid were collected and preserved with 1 ml of metaphosphoric acid (25%, w/v) and sulfuric acid (1%, v/v), and stored at −20°C for the late analysis of VFA profile and NH_3_-N concentration. The fermentation liquid samples from three bottles of each treatment combination were pooled by run, and only the control and medium dose of CB were used for DNA extract. Two samples of 5 ml of fermentation liquid were collected and immediately frozen in liquid nitrogen and preserved at −80°C until DNA extraction.

### Microbial DNA isolation and 16S rRNA gene sequencing

The rumen fluid samples for determining bacterial community were delivered to LC-Sciences (Hangzhou, China) for 16S rRNA gene sequencing. In brief, DNA from fermentation fluid was extracted using the E.Z.N.A. ®Stool DNA Kit (D4015, Omega, Inc., United States) according to the manufacturer’s instructions. The V4–V5 region of the bacteria 16S rRNA gene was amplified by PCR. The amplification was performed using the forward primer 515F (5′-GTGCCAGCMGCCGCGGTAA-3′) and the reverse primer 907R (5′-CCGTCAATTCMTTTRAGTTT-3′). The PCR reactions were performed in triplicate in a 25 μl mixture containing 25 ng of template DNA, 12.5 μl PCR Premix, 2.5 μl of each primer, and PCR-grade water to 25 μl. The PCR reactions were conducted using the following program: (i) initial denaturation at 98°C for 30 s; (ii) 32 cycles of denaturation at 98°C for 10 s, (iii) annealing at 54°C for 30 s, and (iv) extension at 72°C for 45 s; and (v) a final extension step at 72°C for 10 min. The amplified products were detected using 2% agarose gel electrophoresis. The PCR products were purified with AMPure XT beads (Beckman Coulter Genomics, Danvers, MA, United States) and quantified using Qubit (Invitrogen, Carlsbad, United States). The pooled purified libraries were measured for size and quantity with Agilent 2100 Bioanalyzer (Agilent, United States) and further quantified using the Library Quantification Kit for Illumina (Kapa Biosciences, Woburn, MA, United States). Sequencing was performed on NovaSeq PE250 platform.

Paired-end reads were assigned to samples based on their unique barcode and truncated by cutting off the barcode and primer sequence. Paired-end reads were merged using FLASH (version 1.2.8).^1^ Quality filtering on the raw reads was performed under specific filtering conditions to obtain the high-quality clean tags according to the fqtrim (version 0.94).^1^ Chimeric sequences were filtered using Vsearch software (version 2.3.4).^1^ Alpha diversity and beta diversity were calculated by being normalized to the same sequences randomly. Then, according to SILVA (Release 138)^1^ classifier, feature abundance was normalized using the relative abundance of each sample. Alpha diversity is applied in analyzing the complexity of species diversity for a sample through five indices, including Chao1, OTU, Evenness, Shannon, Simpson, and all these indices in the samples were calculated with QIIME2.^1^ Beta diversity was calculated by QIIME2. The blast was used for sequence alignment, and the feature sequences were annotated with the SILVA database for each representative sequence.

### Chemical analyses

Substrate was analyzed for DM (method 930.15), ash and organic matter (method 942.05), and crude protein (method 990.03; N × 6.25) according to AOAC (2005). Neutral detergent fibre was determined as described by Van Soest et al. (1991) with heat-stable α-amylase and sodium sulfite used. Acid detergent fibre was determined according to AOAC (2005), method 973.18. The VFA concentration was determined using a gas chromatograph (GC-2010, Shimadzu) equipped with a capillary column (30 m × 0.32 mm i.d., 1-μm phase thickness, Zebron ZB-FAAP, Phenomenex, Torrance, CA, United States) according to Beauchemin et al. (2003). The NH_3_-N concentrations were determined using the method described by Broderick and Kang (1980).

### Statistical analysis

Data for GP, DMD, and fermentation characteristics were subjected to statistical analysis using the MIXED procedure of SAS (SAS Inst. Inc. Cary, NC, United States) with the model including fixed effects of media pH, a supplemental dose of CB, the interactions, and the random effects of the run. Data for microbiota were analyzed using the MIXED procedure of SAS (SAS Inst. Inc. Cary, NC, United States) with the model including fixed effects of media pH, CB supplementation (control vs. medium dose), the interactions, and the random effects of the run. Tukey’s multiple comparison test was used to examine the significance among treatments within a media pH level for the data of GP, DMD, and fermentation characteristics as well as for the microbiota data. The CONTRAST statement of SAS with linear and quadratic orthogonal contrasts was used to determine the effect of increasing the dose of CB. Differences were declared significant at *p* ≤ 0.05 and trends were discussed at 0.05 < *p* ≤ 0.10 unless otherwise stated.

## Results

### GP, DMD, and rumen fermentation characteristics

There was no interaction between media pH level and dose of CB on GP at 3, 6, 9, and 24 h, but the interaction was significant for the GP at 12 h (*p* = 0.029; Table 2); the GP increased with increasing CB supplementation at 9 h (linear; *p* = 0.011), 12 h (linear; *p* = 0.002), and 24 h (quadratic; *p* < 0.001) at media pH 6.0; whereas it was not affected by increasing CB supplementation except at 24 h (quadratic; *p* = 0.044) at media pH 6.6. Moreover, at media pH 6.6, the GP was greater with supplementation of CB at low and high doses compared with the control and medium doses at 3 h (*p* = 0.022), 12 h (*p* = 0.045), and 24 h (*p* = 0.009) of incubation. Linearly (*p* < 0.001) increasing CB addition also increased DMD without interaction of pH level with the CB supplemental dose. Overall, the GP (*p* < 0.001) and DMD (*p* = 0.008) were greater at media pH 6.6 *vs*. pH 6.0.

**Table 2:** Effects of supplemental doses of *Clostridium butyricum* (CB) on gas production (GP), DM disappearance (DMD), and rumen fermentation characteristics at low and high media pH after 24 h of *in vitro* fermentation.

Doses of CB^2^						*p* value^3^			
Item^1^					SEM
	Control	Low	Medium	High		pH	T	L	Q
**pH 6.0**									
Gas production (ml/g DM)									
3 h	12.8	11.5	13.5	12.8	3.09	<0.001	0.320	0.640	0.988
6 h	26.7	24.0	28.3	27.5	3.18	<0.001	0.073	0.098	0.711
9 h	41.2^b^	40.2^b^	44.1^a^^,^^b^	45.8^a^	3.65	<0.001	0.042	0.011	0.838
12 h	58.2^a^	57.8^a^	62.1^a^^,^^b^	65.5^a^	5.33	<0.001	0.014	0.002	0.752
24 h	121.6^c^	125.8^b^	119.4^c^	140.0^a^	9.02	<0.001	<0.001	<0.001	<0.001
DMD, %	45.5^b^	47.7^b^	47.2^b^	50.9^a^	1.11	0.008	0.010	0.002	0.712
TVFA, mM	54.9	51.1	54.5	52.5	2.30	<0.001	0.059	0.388	0.630
Acetate, mol/100 mol	53.8	53.0	53.3	52.8	0.43	<0.001	0.416	0.177	0.771
Propionate, mol/100 mol	23.4	23.0	22.7	23.2	0.26	<0.001	0.250	0.675	0.055
Butyrate, mol/100 mol	14.4^b^	15.5^a^	15.6^a^	15.7^a^	0.48	0.669	0.042	0.027	0.076
BCVFA	4.40	4.29	4.39	4.27	0.084	< 0.001	0.089	0.100	0.767
A:P ratio	2.30	2.31	2.36	2.28	0.039	<0.001	0.571	0.651	0.248
NH_3_-N, mM	12.7^b^	13.6^a^	14.2^a^	14.0^a^	0.66	<0.001	<0.001	0.001	0.003
**pH 6.6**									
Gas production (ml/g DM)									
3 h	14.7^b^	17.8^a^	15.5^b^	16.2^a^	1.55	<0.001	0.022	0.573	0.167
6 h	30.4	32.8	29.7	32.0	3.02	<0.001	0.194	0.656	0.733
9 h	49.3	53.2	48.4	52.7	2.94	<0.001	0.185	0.403	0.687
12 h	73.4^b^	81.1^a^	73.0^b^	80.0^a^	4.83	<0.001	0.045	0.214	0.835
24 h	142.0^b^	162.5^a^	143.2^b^	159.0^a^	8.79	<0.001	0.009	0.044	0.787
DMD, %	46.9^b^	50.9^a^	48.3^b^	52.8^a^	0.97	0.008	<0.001	<0.001	0.838
TVFA, mM	59.4	60.9	61.0	59.9	5.56	<0.001	0.315	0.857	0.074
Acetate, mol/100 mol	54.2	54.3	54.2	54.1	0.36	<0.001	0.995	0.831	0.879
Propionate, mol/100 mol	22.0	21.7	21.5	22.0	0.55	<0.001	0.193	0.987	0.039
Butyrate, mol/100 mol	14.9	15.3	15.6	15.1	0.54	0.669	0.239	0.645	0.053
BCVFA	4.58	4.60	4.58	4.59	0.096	<0.001	0.995	0.942	0.984
A:P ratio	2.47	2.50	2.53	2.47	0.065	<0.001	0.427	0.833	0.116
NH_3_-N, mM	13.3^b^	14.4^a^	15.3^a^	14.9^a^	0.96	<0.001	0.008	0.009	0.014

abc Means within a row with different superscripts differ (*p* < 0.05).

1 VFA, volatile fatty acids; BCVFA, branched-chain VFA; A:P ratio, acetate: propionate ratio.

2 Control, Low, Medium, and High were supplemented, respectively, with 0, 5 × 10^6^, 1 × 10^6^, and 2× 10^6^ CFU/bottle of *Clostridium butyricum*.

3 pH = pH 6.0 *vs*. 6.6; T = comparison among CB supplemented at Control, Low, Medium and High; L, linear effect of CB addition; Q, quadratic effect of CB addition; pH × T, interaction of pH and Treatments. Interactions: Gas production (12 h), pH × dose, *p* = 0.029.

In comparison with media pH 6.0, the greater (*p* < 0.001) total VFA concentration (averaged 60.3 *vs*. 53.0 mM), molar proportions of acetate (averaged 54.2 *vs*. 53.2%), A:P ratio (averaged 2.49 *vs*. 2.31) and NH_3_-N concentration (averaged 14.5 *vs*. 13.6 mM), and lower (*p* < 0.001) molar proportion of propionate (21.8 *vs*. 23.1%) were observed at media pH 6.6 (Table 2). Furthermore, there was no interaction between pH level and CB supplementation on total VFA concentration, individual VFA profiles, and NH_3_-N concentration. Increasing the supplementation of CB did not affect the total VFA concentrations and the molar proportion of acetate at either media pH 6.0 or pH 6.6. However, increasing the supplemental dose of CB quadratically changed the proportion of propionate (*p* = 0.055 at pH 6.0; *p* = 0.039 at pH 6.6), and linearly increased (*p* = 0.027) the molar proportion of butyrate at pH 6.0. The fermentation pattern, expressed as A:P ratio was not influenced by supplementing CB. It was also observed that the NH_3_-N concentrations linearly increased (pH 6.0, *p* < 0.001; pH 6.6, *p* = 0.008) with increasing supplementation of CB.

### Microbiome diversity analysis

A total of 2,028,448 raw tags with an average of 84,519 raw tags for each sample were generated by 16S rRNA gene sequencing. In addition, a total of 1,854,300 valid tags with an average of 77,263 valid tags for each sample were obtained, and the ratio of valid data to raw data was 89.01–93.00%. The Alpha diversity results showed that the indexes of OTU number (*p* = 0.045) and Chao1 (*p* = 0.044) were lower, whereas Simpson (*p* < 0.001) and Evenness (*p* = 0.005) were greater at media pH 6.0 than pH 6.6 (Table 3). Supplementation of CB increased the indexes of OTU number (*p* = 0.047) and Chao1 (*p* = 0.048), and decreased Simpson (*p* = 0.008) and Evenness (*p* = 0.028) at media pH 6.6 than pH 6.0. All indexes of Alpha diversity were unaffected with supplementation of CB at both media pH levels. The interaction between pH and CB tended to be significant for OTU number (*p* = 0.052) and Chao 1 (*p* = 0.056). A multiple comparison did not clearly show the synergism effect with the combination of high media pH and CB supplementation compared with other treatment combinations.

**Table 3:** Bacterial alpha diversity indexes of fermentation fluid supplemented with CB at low or high media pH after 24 h of *in vitro* incubation.

			Treatments^1^				*p*-value^2^
Index		pH 6.0		pH 6.6	SEM
	Control	CB	Control	CB		pH	T	CB	pH × CB
OTU number	2184^a^	2015^b^	2189^a^	2255^a^	59.2	0.045	0.047	0.371	0.052
Shannon	9.76	9.70	9.69	9.72	0.044	0.483	0.671	0.766	0.341
Simpson	0.997^a^	0.997^a^	0.996^b^	0.996^b^	0.0003	<0.001	0.008	0.866	0.815
Chao1	2189^a^	2019^b^	2197^a^	2261^a^	60.0	0.044	0.048	0.366	0.056
Evenness	0.880^ab^	0.885^a^	0.873^b^	0.872^b^	0.0030	0.005	0.028	0.537	0.438

ab Means within a row with different superscripts differ (*p* < 0.05).

1 CB was supplemented with 1 × 10^6^ CFU/bottle of *Clostridium butyricum*.

2 pH = pH 6.0 *vs*. 6.6; T, comparison among treatments of Control and CB at media pH 6.0 and pH 6.6; CB, Control vs. CB; and pH × CB, interaction of pH and CB.

**Figure 1:**
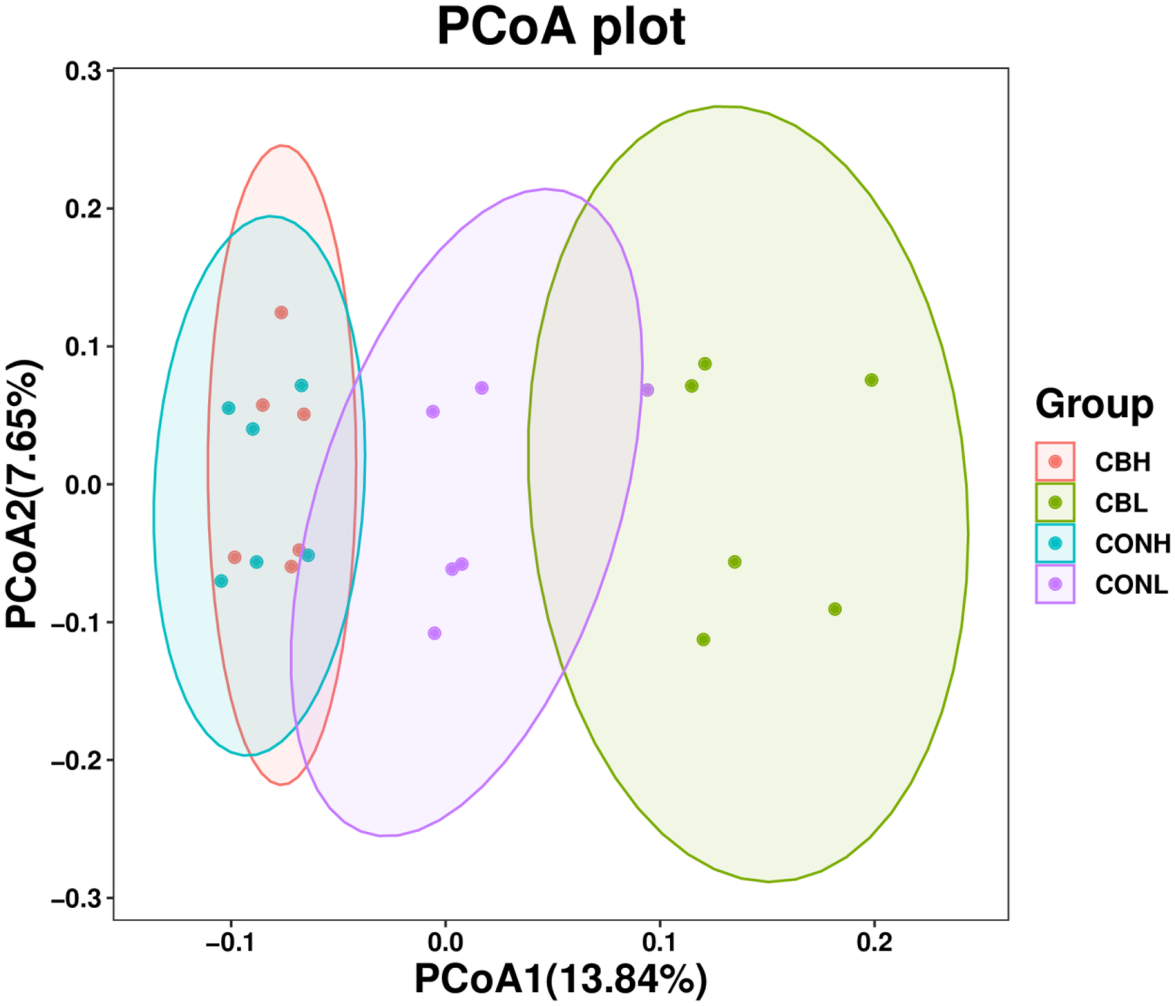
Principal coordinates analysis (PCoA) based on unweighted uniFrac distance matrices of microbiota in ruminal fermentation fluid supplemented with CB at low or high media pH after 24 h of *in vitro* incubation. CONH, control at media pH 6.6; CONL, control at media pH 6.0; CBH, CB supplemented at 1 × 10^6^ CFU/bottle at media pH 6.6; and CBL, CB supplemented at 1 × 10^6^ CFU/bottle at media pH 6.0.

**Figure 2:**
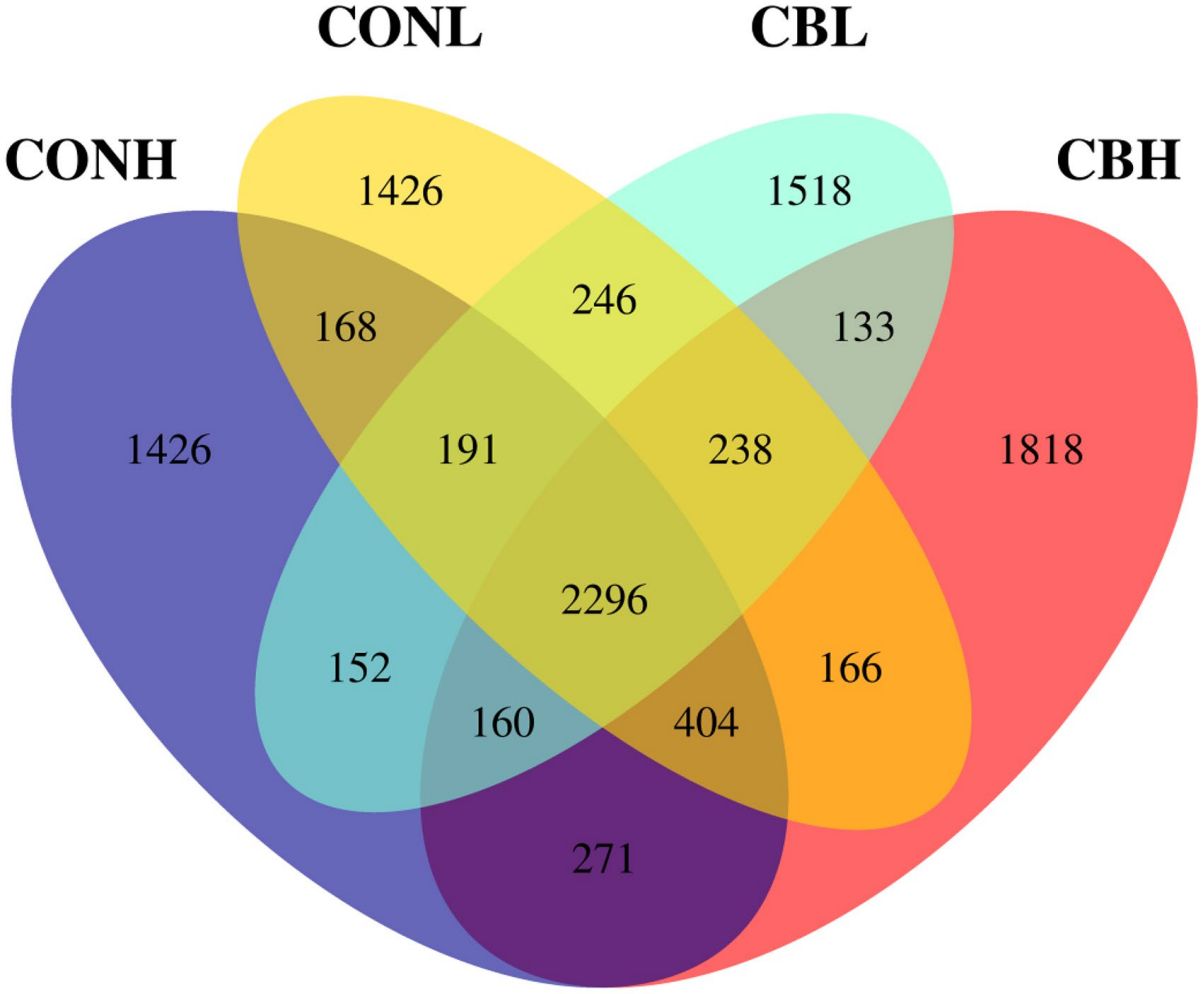
Analysis of OTUs exclusiveness of microbiota in ruminal fermentation fluid supplemented with or without CB at low or high media pH after 24 h of *in vitro* incubation. CONH, control at media pH 6.6; CONL, control at media pH 6.0; CBH, CB supplemented at 1 × 10^6^ CFU/bottle at media pH 6.6; and CBL, CB supplemented at 1 × 10^6^ CFU/bottle at media pH 6.0.

As for the Beta diversity of the bacterial communities in the fermentation culture, the PcoA analysis (unweighted uniFrac) illustrated that the clustering of bacterial microbiota from control and CB overlapped and no clear distinction was observed at media pH 6.6. Differently, clustering of bacterial microbiota of control and CB were distinctly separated from each other at media pH 6.0 (Figure 1). The Venn diagram in the fermentation culture samples showed that the control and CB groups shared 2,971 OTUs, and had 2,164 and 1,963 exclusive OTUs (respectively) media pH 6.0 (Figure 2). Moreover, there were 3,131 shared OTUs, and 1,937 and 2,355 exclusive OTUs for the control and CB groups at media pH 6.6.

### Bacterial community

The relative abundances of the 10 most abundant bacterial phyla were detected in the fermentation culture of control and CB (medium dose) groups (Table 4). The relative abundances of *Bacteroidota* (*p* < 0.001), *Bacteroidetes* (*p* = 0.004), and *Desulfobacterota* (*p* = 0.001) were lower, but *Firmicutes* (*p* = 0.026), *Proteobacteria* (*p* = 0.035), *Verrucomicrobiota* (*p* < 0.001), and *Cyanobacteria* (*p* < 0.001) were greater at media pH 6.6 than pH 6.0. At media pH 6.0, the relative abundances of *Bacteroidota* (*p* < 0.001), *Bacteroidetes* (*p* = 0.031), and *Desulfobacterota* (*p* = 0.001) were greater, whereas *Verrucomicrobiota* (*p* < 0.001), *Spirochaetota* (*p* < 0.001), and *Cyanobacteria* (*p* < 0.001) were lower with supplementation of CB compared with media pH 6.6. Supplementation of CB decreased the relative abundances of *Proteobacteria* (*p* < 0.001) and *Verrucomicrobiota* (*p* = 0.004), and increased *Desulfobacterota* (*p* = 0.023) and *Cyanobacteria* (*p* = 0.003). Interaction (*p* < 0.001) between pH and CB were observed for *Spirochaetota.* A multiple comparison among four means did not find a consistent optimal pH and CB combination.

**Table 4:** The relative abundance (%) of bacterial phylum in fermentation fluid supplemented with CB and without CB (Control) at low or high media pH after 24 h of *in vitro* incubation.

		Treatments^1^						*p* value^2^
	pH6.0		pH6.6		SEM
Phylum	Control	CB	Control	CB		pH	T	CB	pH × CB
*Bacteroidota*	47.5^a^	48.2^a^	39.3^b^	40.8^b^	1.26	<0.001	<0.001	0.375	0.750
*Firmicutes*	31.4	33.1	34.9	35.5	1.23	0.026	0.110	0.357	0.670
*Proteobacteria*	8.33^a^	7.20^b^	8.90^a^	7.64^b^	0.223	0.035	<0.001	<0.001	0.785
*Verrucomicrobiota*	4.50^b^	3.19^c^	7.10^a^	6.16^a^	0.341	<0.001	<0.001	0.004	0.595
*Bacteroidetes*	2.75^a^ ^b^	2.90^a^	2.26^bc^	2.22^c^	0.180	0.004	0.031	0.791	0.609
*Spirochaetota*	1.23^b^	0.46^c^	3.06^a^	3.23^a^	0.116	<0.001	<0.001	0.014	<0.001
*Desulfobacterota*	0.87^b^	1.29^a^	0.65^b^	0.73^b^	0.102	0.001	0.001	0.023	0.114
*Cyanobacteria*	0.62^b^	0.43^c^	0.86^a^	0.72^a^ ^b^	0.049	<0.001	<0.001	0.003	0.555
*Synergistota*	0.57	0.58	0.57	0.61	0.046	0.656	0.881	0.582	0.708
*Unclassified*	0.55^a^	0.46^b^	0.49^b^	0.59^a^	0.033	0.185	0.017	0.852	0.003

abc Means within a row with different superscripts differ (*p* < 0.05).

1 CB was supplemented with 1 × 10^6^ CFU/bottle of *Clostridium butyricum*.

2 pH = pH 6.0 *vs*. 6.6; T, comparison among treatments of Control and CB at media pH 6.0 and pH 6.6; CB, Control *vs*. CB; and pH × CB, interaction of pH and CB.

As shown in Table 5, the relative abundances of 27 predominant bacterial genera (>1% at least in one group) were detected in the fermentation culture. There were interactions for *Christensenellaceae_R-7_group* (*p* = 0.001), *NK4A214_group* (*p* = 0.003), *Succinivibrionaceae_UCG-002* (*p* = 0.030), and *Muribaculaceae_unclassified* (*p* < 0.001). Furthermore, adding CB increased *Christensenellaceae_R-7_group* (*p* = 0.001), *NK4A214_group* (*p* = 0.003), and *Muribaculaceae_unclassified* (*p* < 0.001), and decreased *Succinivibrionaceae_UCG-002* (*p* = 0.002) at media pH 6.0, but they were not affected at media pH 6.6. In comparison with media pH 6.6, the relative abundances of *Prevotella* (*p* < 0.001), *Rikenellaceae_RC9_gut_group* (*p* < 0.001), *Christensenellaceae_R-7_group* (*p* < 0.001), *NK4A214_group* (*p* < 0.001), *Ruminococcus* (*p* = 0.040), *Muribaculaceae_unclassified* (*p* < 0.001), *Prevotellaceae_UCG-001* (*p* < 0.001), *Clostridia_UCG-014_unclassified* (*p* < 0.001), *Bacteroidales_unclassified* (*p* < 0.001), *Bacteroidetes_unclassified* (*p* < 0.001) and *Desulfovibrio* (*p* = 0.001) were greater, whereas *CAG-352* (*p* < 0.001), *WCHB1-41_unclassified* (*p* < 0.001), *Bacteroidales_RF16_group_unclassified* (*p* = 0.003), *Firmicutes_unclassified* (*p* = 0.001), *Ruminococcaceae_unclassified* (*p* < 0.001), *Treponema* (*p* < 0.001), and *UCG-010_unclassified* (*p* < 0.001) were lower at media pH 6.0. The relative abundances of *Prevotella* (*p* < 0.001), *Rikenellaceae_RC9_gut_group* (*p* < 0.001), *Christensenellaceae_R-7_group* (*p* < 0.001), *NK4A214_group* (*p* < 0.001), *Muribaculaceae_unclassified* (*p* < 0.001), *Prevotellaceae_UCG-001* (*p* = 0.002), *Clostridia_UCG-014_unclassified* (*p* < 0.001), *Bacteroidales_unclassified* (*p* < 0.001), *Acinetobacter* (*p* = 0.008), *Bacteroidetes_unclassified* (*p* < 0.001), *and Desulfovibrio* (*p* = 0.001) were greater, whereas *CAG-352* (*p* < 0.001), *WCHB1-41_unclassified* (*p* < 0.001)*, Bacteroidales_RF16_group_unclassified* (*p* = 0.005), *Firmicutes_unclassified* (*p* = 0.005), *Ruminococcaceae_unclassified* (*p* < 0.001), *Treponema* (*p* < 0.001), and *UCG-010_unclassified* (*p* < 0.001) were lower with supplementation at media pH 6.0 than pH 6.6. The CB supplementation increased the relative abundances of *Rikenellaceae_RC9_gut_group* (*p* = 0.002), *Clostridia_UCG-014_unclassified* (*p* = 0.042), *Bacteroidales_unclassified* (*p* = 0.047) and *Bacteroidetes_unclassified* (*p* = 0.028), and *Desulfovibrio* (*p* = 0.022), and decreased *WCHB1-41_unclassified* (*p* = 0.007), and *Bacteroidales_RF16_group_unclassified* (*p* = 0.030) and *Prevotellaceae_UCG-003* (*p* = 0.036). A multiple comparison among four means was unable to conclude a consistent optimal pH and CB combination on the relative abundance of bacterial genus.

**Table 5:** The relative abundance (%) of bacterial genus (>1% at least in one group) in ruminal fermentation fluid supplemented with CB and without CB (control) at low or high media pH after 24 h of vitro incubation.

	Treatments^1^					SEM	*p* value^2^
	pH6.0	pH6.6
Genus	Control	CB	Control	CB	pH	T	CB	pH × CB
*Prevotella*	13.92^a^	13.44^a^	10.87^b^	10.69^b^	0.477	<0.001	<0.001	0.496	0.754
*Rikenellaceae_RC9_gut_group*	11.92^b^	13.94^a^	8.48^c^	9.23^c^	0.411	< 0.001	<0.001	0.002	0.113
*F082_unclassified*	7.00	7.35	6.08	6.72	0.488	0.127	0.326	0.317	0.768
*CAG-352*	4.91^b^	1.76^c^	9.21^a^	9.54^a^	0.711	<0.001	<0.001	0.061	0.024
*WCHB1-41_unclassified*	3.77^b^	2.77^c^	5.87^a^	5.02^a^	0.303	<0.001	<0.001	0.007	0.811
*Bacteroidales_RF16_group_unclassified*	3.22^a^	1.70^b^	4.29^a^	3.74^a^	0.485	0.003	0.005	0.030	0.275
*Christensenellaceae_R-7_group*	2.73^b^	4.53^a^	2.12^b^	2.27^b^	0.217	<0.001	<0.001	<0.001	0.001
*NK4A214_group*	2.79^b^	4.26^a^	2.17^c^	2.19^bc^	0.203	<0.001	<0.001	0.002	0.003
*Prevotellaceae_UCG-003*	3.03^a^	2.60^b^	2.63^b^	2.60^b^	0.108	0.063	0.020	0.036	0.065
*Bacteroidales_BS11_gut_group_unclassified*	2.61	2.30	2.56	2.82	0.333	0.494	0.747	0.941	0.399
*Succinivibrionaceae_UCG-002*	2.87^a^	1.84^c^	2.40^a^ ^b^	2.21^bc^	0.180	0.770	0.006	0.003	0.030
*Firmicutes_unclassified*	2.12^a^ ^b^	1.55^b^	2.63^a^	2.58^a^	0.212	0.001	0.005	0.138	0.214
*Ruminococcus*	2.08	2.32	1.79	1.67	0.215	0.040	0.171	0.789	0.425
*Muribaculaceae_unclassified*	1.73^b^	2.91^a^	1.01^c^	1.30^c^	0.110	<0.001	<0.001	<0.001	<0.001
*Ruminococcaceae_unclassified*	1.72^a^	1.06^b^	1.92^a^	2.04^a^	0.133	<0.001	<0.001	0.056	0.010
*Prevotellaceae_UCG-001*	1.96^a^	2.00^a^	1.32^b^	1.33^b^	0.142	<0.001	0.002	0.849	0.912
*Succiniclasticum*	1.47	2.24	1.32	1.28	0.272	0.056	0.072	0.192	0.153
*Clostridiales_Family_IV._Incertae_Sedis_unclassified*	1.51	1.42	1.68	1.60	0.110	0.125	0.389	0.458	0.982
*Clostridia_UCG-014_unclassified*	1.50^b^	1.78^a^	1.09^c^	1.13^c^	0.075	<0.001	<0.001	0.042	0.120
*Treponema*	0.71^b^	0.19^c^	2.17^a^	2.32^a^	0.101	<0.001	<0.001	0.085	0.005
*UCG-010_unclassified*	1.16^b^	1.02^b^	1.36^a^	1.38^a^	0.049	<0.001	<0.001	0.263	0.119
*Sutterella*	0.97	1.05	1.06	1.04	0.129	0.755	0.962	0.823	0.716
*p-251-o5_unclassified*	1.07	0.93	0.93	1.08	0.070	0.940	0.251	0.940	0.051
*Bacteroidales_unclassified*	1.01^a^	1.14^a^	0.75^b^	0.82^b^	0.045	<0.001	<0.001	0.047	0.551
*Acinetobacter*	0.77^bc^	1.08^a^	0.91^a^ ^b^	0.70^c^	0.076	0.101	0.008	0.498	0.002
*Bacteroidetes_unclassified*	0.93^b^	1.11^a^	0.65^c^	0.70^c^	0.049	<0.001	<0.001	0.028	0.193
*Desulfovibrio*	0.69^b^	1.05^a^	0.51^b^	0.57^b^	0.085	0.001	0.001	0.022	0.088

abc Means within a row with different superscripts differ (*p* < 0.05).

1 CB was supplemented with 1 × 10^6^ CFU/bottle of *Clostridium butyricum*.

2 pH = pH 6.0 *vs*. 6.6; T, comparison among treatments of Control and CB at media pH 6.0 and pH 6.6; CB, Control *vs*. CB; and pH × CB, interaction of pH and CB.

## Discussion

### Gas production, DMD, and fermentation characteristics

The ruminal pH dynamics and pH maximum occurred just before feeding, and the pH minimum was detected approximately 6–7 h after feeding. The mean pH ranged from 5.89 to 6.63 in the rumen when the dairy cows were fed a corn-silage based total mixed ration (Palmonari et al., 2010). Russell and Wilson (1996) suggested that at ruminal pH below 6.0 could be considered a threshold value for subacute rumen acidosis. Therefore, the target media pH was selected at 6.0 and 6.6 for low and high media pH, respectively, also in accordance with the guidelines of our previous *in vitro* studies with pH 5.8 and 6.5 for low and high media pH (Jiao et al., 2019, 2021). In these studies, the low media pH was set at 5.8 because the high-grain substrate (>85%, DM basis) was used and lower pH was expected. In the present study, the final media pH was 6.17 and 6.69, which were close to our target media low pH of 6.0 and high pH of 6.6, indicating high buffering of fermentation media despite fermentation acids produced from the substrate. The overall greater GP, DMD, and total VFA concentration with pH 6.6 than pH 6.0 in the present study confirmed the increased microbial activity and feed degradability with high rumen pH. In fact, the higher proportion of acetate and lower proportion of propionate that resulted in a higher A:P ratio with media pH 6.6 vs. 6.0 suggested an improvement in fibre digestion. The results were consistent with the experiment conducted by Jiao et al. (2019), who observed an increase in the GP, DMD, total VFA concentration, and A:P ratio from low (5.8) to high media pH (6.5) in batch culture using a diet containing 60% forage. In addition, our outcomes revealed that phylum *Proteobacteria* involved in fibre digestion (Qiu et al., 2020; Wei et al., 2022) and its relative abundance was decreased at media pH 6.0 compared with pH 6.6, indicating an inhibition effect of low media pH on fibre digestion. Thus, the varied results of GP, DMD and fermentation characteristics could be attributed to the inhibition of growth of phylum *Proteobacteria* at low media pH *vs*. high media pH.

Cai et al. (2021a) reported that supplementation of CB at 2 × 10^4^ and 4 × 10^4^ CFU/bottle increased the *in vitro* digestibilities of DM, NDF, and ADF using rumen inoculum from goats fed a diet containing 56% forage. Our study presents similar results and detected a linear increase in the accumulative GP and DMD at 24 h after increasing the supplemental dose of CB at either media pH 6.0 or pH 6.6. The improved digestibility by adding CB may be explained by its ability to provide short-chain fatty acids, nutritional factors such as enzymes (exo-pectate lyase, pectin methylesterase, and endopectatelyase) and vitamins B and E that could improve the activities of cellulolytic enzymes (e.g., avicelase, CMCase, cellobiase, and xylanase) in the rumen (Cai et al., 2021a,b). Furthermore, the increased butyrate proportion due to CB addition at media pH 6.0 (linear) and pH 6.6 (quadratic) suggested in this study could be partially explained by the butyrate produced by CB. In addition, although the proportion of propionate quadratically changed after increasing the supplemented CB at both media pH levels, its differences among treatments were minimal from a biological perspective. Li et al. (2021) reported increased proportions of propionate and butyrate without changing the proportion of acetate in Holstein heifers supplemented with CB at 2 × 10^8^ CFU/kg dietary DM. Cai et al. (2021b) demonstrated an increase in acetate concentration with dietary supplementation of CB (1 × 10^8^ CFU/g) at 0.05% of the basal diet in heat-stressed goats. The inconsistent effects of CB addition on the VFA profiles found in different studies may be attributed to the different supplemental doses, animal species, animal health status, or experimental approaches (e.g., *in vitro* vs. *in vivo*) used in each study. Heat-stressed animals often decrease feed intake, which may positively affect fibre digestibility (i.e., an increase of acetate concentration) due to potentially longer rumen retention. Moreover, the increased NH_3_-N concentration by supplementation of CB regardless of media pH levels is consistent with the results presented by Cai et al. (2021b) who found an increase of NH_3_-N concentration with supplementation of CB in the diet of goats. The results suggested promotion of protein degradation as increasing DMD was observed after CB supplementation, but a better microbial protein synthesis was unlikely to happen because the GP and VFA concentration were not lower, and NH_3_-N concentration kept surging (Blümmel and Ørskov, 1993).

### Ruminal bacterial microbiota

Compared with the moderate high forage diet, feeding ruminant with a high concentrate diet would affect fermentation characteristics and the bacterial community, leading to decreased bacterial alpha diversity indexes and rumen pH (Hook et al., 2011; Ma et al., 2021). Zhang et al. (2017) found that the rumen pH linearly decreased when the concentrate level was gradually increased from 20 to 80% in the diet of Holstein heifers, resulting in the decreased bacterial alpha diversity indexes of observed species, PD whole tree, and Pielou in the rumen at lower pH. Consistent with previous studies, our results showed that the bacterial alpha diversity indexes measured as OTU number and Chao 1 increased at media pH 6.6 compared with pH 6.0. Moreover, the PCoA unweighted uniFrac analysis showed that the control groups significantly differed between media pH 6.0 and pH 6.6. The current results of bacterial diversity could be explained by the inhibition of some gram-negative bacteria which were sensitive to the low rumen pH (Khafipour et al., 2009). However, it is important to acknowledge that data on CB supplementation on rumen bacterial diversity is limited. It has been reported that the bacterial diversity was not affected by probiotic supplementation, such as lactic acid bacteria in the diet of sheep (Mani et al., 2021) or active dried yeast in the diet of dairy cows (AlZahal et al., 2017).

The predominant bacterial phyla in the rumen were *Bacteroidota* and *Firmicutes* regardless of media pH levels or CB supplementation involved in the present study. Our result are aligned with the findings presented by Li et al. (2022) and Liu et al. (2022) who reported that *Bacteroidota* and *Firmicutes* were the dominant bacteria in the rumen of dairy cows. We also identified that 8 in 10 abundances of bacterial phyla and 18 in 27 abundances of bacterial genus in the culture media were affected by media pH levels, which may be partly explained by the death and lysis of Gram-negative bacteria caused by low media pH (Nagaraja and Titgemeyer, 2007). Similar studies have reported that the increased *Firmicutes:Bacteroidota* ratio in the rumen would favor the capacity for harvesting energy from the diet and has been linked to milk fat yield in dairy cows (Lima et al., 2014; Tong et al., 2022). The increased relative abundance of *Firmicutes* and decreased *Bacteroidota* at media pH 6.6 increased *Firmicutes:Bacteroidota* ratio compared with media pH 6.0, which may contribute to the greater DMD and total VFA concentration at media pH 6.6 than pH 6.0 in the present study.

Several studies demonstrated that supplementation of CB in the diets had an effect on the modulation of bacterial communities in the feces of weaned piglets (Liang et al., 2021), intestine of chickens (Huang et al., 2019), or rumen and rectum of goats (Zhang et al., 2022). In the present study, the relative abundances of five phyla (10 in total) and 12 genera (27 in total) were changed with the supplementation of CB, which may be due to the nutritional factors that change with supplementation (Li et al., 2021). Although the relative abundance of *Bacteroidota* did not alter, the *Rikenellaceae_RC9_gut_group*, which belongs to this phylum, increased with CB supplementation. Previous studies reported that *Rikenellaceae_RC9_gut_group* contains the genes for secreting cellulase and hemicellulase, resulting in an improvement of energy gain from the fibre degradation (Escobar et al., 2014; Fan et al., 2020). In addition, the surging relative abundance of *Christensenellaceae_R-7_group* with increased the CB supplementation may explain the elevated DMD in the current study. In fact, Huang et al. (2021) reported that the increased abundance of *Christensenellaceae_R-7_group* favored rumen development by increasing nipple width, epithelial thickness, and stratum corneum thickness and contributed to the absorption and digestion of nutrients in the rumen. Moreover, as cellulose-degrading bacteria, the increased abundance of *NK4A214_group* by supplementation of CB suggested an improvement of cellulose degradation that explained the increased DMD (Guo et al., 2020; Yu et al., 2020). It is worth mentioning that there were interactions between media pH and CB supplementation for some species of bacteria either in phylum (2/10) and genus (9/27), indicating that the effects of CB could be different when rumen pH differs, particularly when the response to CB has different direction depending on the media pH levels. Therefore, it can be speculated that the mode of action of CB at different pH levels may be different for some bacteria which are sensitive to media pH levels.

## Conclusion

This study showed that the GP, DMD, total VFA concentrations, and A:P ratio increased by elevating media pH from 6.0 to 6.6, indicating stimulation of rumen fermentation, which may be partially due to changes in their bacterial diversity indexes and relative abundance associated with fibre degradation at phylum or genus level. Linearly increasing supplementation of CB increased DMD and quadratically changed the proportions of propionate and butyrate and NH_3_-N concentration at both media pH levels, revealing the dose-dependent traits of the CB effect. The supplementation of CB affected the rumen microbiota at either phylum or genus level, and the increased abundances of *Rikenellaceae_RC9_gut_group*, *Christensenellaceae_R-7_group*, and *NK4A214_group* were considered favorable to cellulose degradation, therefore increasing the DMD of the diet containing moderate high forage. Furthermore, the interaction of the effects of CB with media pH level on the abundance of microbiota is warranted to search for the optimum rumen pH to maximize CB activity.

## Data availability statement

The datasets presented in this study can be found in online repositories. The names of the repository/repositories and accession number(s) can be found at: https://www.ncbi.nlm.nih.gov/, PRJNA844474.

## Author contributions

MZ and PJ conceived and designed the experiments. MZ, GL, XZ, XL, SL, and XW performed the experiments. MZ, WY, and YY analyzed the data. MZ and PJ contributed to the writing of the manuscript. All authors reviewed and approved the manuscript.

## Funding

This research was supported by the Natural Science Foundation of Heilongjiang Province (YQ2021C018), Postdoctoral Foundation of Heilongjiang Province (LBH-Z21100 and LBH-Z21042), Young Talents Project of Northeast Agricultural University (19QC42), and the University Nursing Program for Young Scholars with Creative Talents in Heilongjiang Province (UNPYSCT-2020106).

## Conflict of interest

The authors declare that the research was conducted in the absence of any commercial or financial relationships that could be construed as a potential conflict of interest.

## Publisher’s note

All claims expressed in this article are solely those of the authors and do not necessarily represent those of their affiliated organizations, or those of the publisher, the editors and the reviewers. Any product that may be evaluated in this article, or claim that may be made by its manufacturer, is not guaranteed or endorsed by the publisher.

## Footnotes

^1^ http://ccb.jhu.edu/software/FLASH/

^1^ http://ccb.jhu.edu/software/fqtrim/

^1^ https://github.com/torognes/vsearch

^1^ https://www.arbsilva.de/documentation/release138/

^1^ https://qiime2.org/
